# ﻿New species and new combinations in the genus *Paraisaria* (Hypocreales, Ophiocordycipitaceae) from the U.S.A., supported by polyphasic analysis

**DOI:** 10.3897/mycokeys.100.110959

**Published:** 2023-11-13

**Authors:** Richard M. Tehan, Connor B. Dooley, Edward G. Barge, Kerry L. McPhail, Joseph W. Spatafora

**Affiliations:** 1 Department of Pharmaceutical Sciences, College of Pharmacy, Oregon State University, Corvallis, Oregon 97331, USA; 2 Department of Chemistry and Biochemistry, Utica University, Utica, New York 13502, USA; 3 Department of Botany and Plant Pathology, College of Agricultural and Life Sciences, Oregon State University, Corvallis, Oregon 97331, USA; 4 Seed Testing Laboratory, Idaho State Department of Agriculture, Boise, ID 83712, USA

**Keywords:** Ascomycota, chemotaxonomy, Cicada, Cordyceps, Cyphoderris, entomopathogen, Ophiocordyceps, Prionus

## Abstract

Molecular phylogenetic and chemical analyses, and morphological characterization of collections of North American *Paraisaria* specimens support the description of two new species and two new combinations for known species. *P.cascadensis***sp. nov.** is a pathogen of *Cyphoderris* (Orthoptera) from the Pacific Northwest USA and *P.pseudoheteropoda***sp. nov.** is a pathogen of cicadae (Hemiptera) from the Southeast USA. New combinations are made for *Ophiocordycepsinsignis* and *O.monticola* based on morphological, ecological, and chemical study. A new cyclopeptide family proved indispensable in providing chemotaxonomic markers for resolving species in degraded herbarium specimens for which DNA sequencing is intractable. This approach enabled the critical linkage of a 142-year-old type specimen to a phylogenetic clade. The diversity of *Paraisaria* in North America and the utility of chemotaxonomy for the genus are discussed.

## ﻿Introduction

*Paraisaria* is an asexual morph-typified genus of entomopathogenic fungi, originally described by Samson and Brady in 1983, characterized by synnemata with verticillately-branched conidiophores and flask-shaped sympodially proliferating phialides (Samson and Brady 1983). These asexual morphs were derived from larvae (Delacroix 1893) and from cultured isolates of the sexual morphs of species in the genus *Cordyceps* (Samson and Brady 1983; Li et al. 2004), which were later transferred to *Ophiocordyceps* (Sung et al. 2007). *Paraisaria* was later proposed for suppression, along with four other genera then in use, in favor of recognizing a broad concept of *Ophiocordyceps* (Quandt et al. 2014). This limited the number of new combinations required to accommodate 1F1N rules following the abolition of the dual system of nomenclature in which sexual states and asexual states of fungi were classified separately. In molecular analyses, *Paraisaria* has been recovered as a distinct monophyletic clade, being referred to as the “gracilis subclade” within the ravenelii subclade” of *Ophiocordyceps* by Sanjuan et al. (2015). *Paraisaria* was ultimately resurrected in 2019, segregated from *Ophiocordyceps*, and amended to include sexual morphology (Mongkolsamrit et al. 2019). *Paraisaria* species possess distinctive sexual morphs characterized by a globose fertile terminal portion of the stroma with immersed perithecia. Thus, *Paraisaria* constitutes a distinct, and robustly monophyletic clade deserving a unique genus classification, though its segregation from *Ophiocordyceps* rendered *Ophiocordyceps* into several paraphyletic clades. Ultimately, a comprehensive analysis of *Ophiocordyceps* sensu Sung et al. (2007), is needed to establish robust generic concepts and restore global monophyly. A major sticking point for this action is the uncertain placement of the type of *Ophiocordyceps*, *O.blattae*, among the paraphyletic subclades of *Ophiocordyceps*.

In North America, *Paraisaria* species are unique among most *Cordyceps* sensu lato in that they form fruiting bodies in the spring, whereas most other insect pathogens fruit in the summer, fall, or winter months, which is evident in herbarium records on MycoPortal (MycoPortal 2023) and observations on the community science platform iNaturalist (https://www.inaturalist.org/projects/north-american-cordyceps-sensu-lato). Most *Paraisaria* species, and thus far, all known *Paraisaria* species occurring in North America, form fruiting bodies on subterranean insect hosts.

Some of the insect hosts of *Paraisaria* species are sought as food and their contamination by *Paraisaria* species could pose a human health concern. Doan et al. (2017) reported a series of poisonings and one fatality in Southern Vietnam, among people who had consumed cicadae infected with a fungus identified as *Paraisariaheteropoda* (=*Cordycepsheteropoda*, *Ophiocordycepsheteropoda*), between 2008 and 2015. The toxicity was attributed to the presence of mycotoxins in the otherwise edible cicadae, and the toxic agent was putatively identified as ibotenic acid. The potential role of entomopathogenic fungi in causing food-borne mycotoxin poisonings underscores the need to describe the biological and chemical diversity present in this group of fungi.

In addition to their impact on human and animal health, fungal natural products can be highly useful phenotypic characters for taxonomic purposes. Chemical fingerprints can be used to identify chemical families that constitute a generic chemotype for a taxonomic group, and also unique suites of compounds within a chemical family can be used to resolve species. For example, Cedeño-Sanchez et al. (2023) profiled chemical extracts from stromata to characterize and distinguish species and genera in the family Hypoxylaceae.

Only two studies (Krasnoff et al. 2005; Umeyama et al. 2011) have reported a total of five natural products from *Paraisaria* species, both of which investigated fungi identified as the cicada pathogen, *Paraisariaheteropoda*. A third study reports leucinostatin analogs from an organism reported as *Ophiocordycepsheteropoda* (=*Parasiaraheteropoda*) (Kil et al. 2020), but which is evidently a *Purpureocillium* species based on ITS phylogeny and chemotaxonomy. Doan et al. (2017) also report the amino acid, ibotenic acid from this species, but no analytical chemistry data are presented to confirm this. There are currently no published genome sequences available to mine the specialized metabolic potential of *Paraisaria* species, although the sequenced genomes of other Ophiocordycipitaceae species display a familial trend of high biosynthetic capacity for specialized metabolites. The first chemical study of a member of this genus resulted in the discovery of the new 8-residue antimicrobial peptaibiotics, cicadapeptins I and II, which possessed a unique two consecutive 4-hydroxyproline residues at the N-terminus (Krasnoff et al. 2005). The known antifungal and immunosuppressant sphingosine analog, myriocin was also isolated in this study. Heteropodamides A and B are N-methylated cyclic heptapeptides reported as cytotoxins from *P.heteropoda* (Umeyama et al. 2011). Their absolute structures are yet to be determined. The further discovery of *Paraisaria* species and their natural products presents fertile grounds for investigation.

In the course of ongoing investigations for the discovery of biologically active natural products from *Paraisaria* species (Tehan 2022), it became critical to perform a taxonomic analysis of North American *Paraisaria* to better understand the biological diversity present in this group. In this study, we examined 29 recent collections of *Paraisaria* to investigate the diversity of North American *Paraisaria*. We also analyzed the type collections of *Ophiocordycepsinsignis* and *O.monticola*, both of which were anticipated to belong in *Paraisaria* based on morphological description, ecology, and phenology. One phylogenetically informative DNA sequence was afforded from the 87-year old *O.monticola* specimen. The 142-year old *O.insignis* type did not permit successful DNA sequencing, however, chemical analysis of the newly characterized paraisariamide family of compounds by LC-HRMS provided robust support for the combination of both species into *Paraisaria*, as well as the correct identification of a species of importance to human health, as *P.insignis*. This study provides a novel framework for the use of minimally destructive chemical analysis in taxonomic assessment of type specimens where DNA sequencing is not possible. The combined analysis of molecular data, morphology, ecology, phenology, and chemical data support the circumscription of two new species and two new combinations, and provides an initial overview of the diversity of American *Paraisaria* species.

## ﻿Materials and methods

### ﻿Specimens and isolates

Twenty nine new collections of *Paraisaria* specimens and their insect hosts were examined. Macroscopic characters were examined from fresh stromata, and microscopic characters were examined from fresh and dried stromata, including ascospores discharged from fresh stromata when possible and sections of dried specimens. Colors are in general terms of the senior author. Specimens are deposited in the Oregon State University Herbarium mycological collection. Culture isolates of fungi were made from tissue dissected from the context of stromata, placed on PDA with 50 µg/ml ampicillin and 100 µg/ml streptomycin, or from ascospores germinated on PDA. Agar plugs were taken from outgrowth of stromatic tissue and subcultured onto PDA and CMA at 20 °C. Cultures are deposited at the USDA ARS Collection of Entomopathogenic Fungal Cultures (ARSEF).

### ﻿Morphological observations

Fruiting bodies were examined for morphological measurements using a Vernier caliper (Fowler). Sections of ascogenous tissue were mounted in lactophenol cotton blue, 5% KOH, or distilled water, and microanatomical characters were examined with light microscopy using a Leica DM2500. Twenty each, perithecia, asci, and part-spores were measured at magnifications of 10×, 20×, 40×, 63×, or 100×.

### ﻿DNA extraction and sequencing

DNA was extracted from the ascogenous portion of dried stromata, ground with mortar and pestle in CTAB buffer (1.4 M NaCl, 100 mM Tris–HCl pH 8.0, 20 mM EDTA pH 8.0, 2% CTAB w/v) and processed following the method of Kepler et al. (2012). Samples were extracted with 25:24:1 phenol:chloroform:isoamyl alcohol, (affymetrix), and DNA was precipitated with 3 M sodium acetate (pH 5.2) and 95% ethanol. PCR amplification was performed on the Internal Transcribed Spacer (ITS), amplified using ITS4 and ITS5 primers (White et al. 1990). Alternatively, ITS1F (Gardes and Bruns 1993) was used as a forward primer for samples where ITS4 did not work. For samples in which amplification of the ITS region did not succeed, individual amplification of the ITS1 and ITS2 loci was attempted using primer sets ITS5 and ITS2 (White et al. 1990) for the ITS1 locus, and ITS3 (White et al. 1990) and ITS4 for the ITS2 locus. Nuclear small subunit (nucSSU) was amplified using nucSSU131 and NS24 (Kauff and Lutzoni 2002), nuclear large subunit (nucLSU) using LROR (Rehner and Samuels 1994) and LR7 (Vilgalys and Hester 1990), subunit 1 of RNA polymerase II (RPB1) using RPB1-A_f_ and *RPB*1-6R1asc (Hofstetter et al. 2007). Alternatively, CRPB-1 (Castlebury et al. 2004) was used as a forward primer for samples where RPB1-A_f_ did not work. Elongation factor 1α (EF-1α) was amplified using 983F and 2218R (Castlebury et al. 2004). PCR was performed with an iCycler (Bio- Rad, USA), with a total of 20 μl reaction mixture containing 1× PCR Buffer (Promega), 1× TBTpar prepared as in Samarakoon et al. (2013), 2.5 mM MgCl_2_, 0.5 µM each forward and reverse primers, 200 µM of each of the four dNTPs, and 0.5 U Taq polymerase. For ITS, SSU, LSU, and TEF, the PCR thermal cycle consisted of an initial 1 min denaturation at 95 °C; 34 cycles of 30 s at 94 °C, 1 min at 52 °C, 1.5 min at 72 °C, and a termination with an elongation 7 min at 72 °C. For RPB1 and RPB2, the PCR thermal cycle consisted of an initial 1.5 min denaturation at 95 °C; 39 cycles of 30 s at 94 °C, 1 min at 47 °C, 2 min at 72 °C, and a termination with an elongation 4 min at 72 °C. Sequencing was performed by the Sanger method at the Center for Quantitative Life Sciences at Oregon State University. The sequences obtained in this study were deposited to GenBank (Table 1).

**Table 1.** Sequences used in phylogenetic tree construction.

Species	Code	Host	ITS	SSU	LSU	EF1a	RPB1	RPB2	Reference
*Cordycepskyushuensis*	EFCC 5886	Lepidoptera	—	EF468960	EF468813	EF468754	EF468863	EF468917	Sung et al. 2007
*Cordycepsmilitaris*	OSC.93623	Lepidoptera	JN049825	AY184977	AY184966	DQ522332	DQ522377	—	Kepler et al. 2017
*Drechmeriabalanoides*	CBS 250.82	Nematoda	MH861495	AF339588	AF339539	DQ522342	DQ522388	DQ522442	Vu et al. 2019
*Drechmeriasinensis*	CBS 567.95	Nematoda	MH862540	AF339594	AF339545	DQ522343	DQ522389	DQ522443	Spatafora et al. 2007
*Harposporiumanguillulae*	ARSEF 5407	Nematoda	—	—	AY636080	—	—	—	Chaverri et al. 2005
*Harposporiumhelicoides*	ARSEF 5354	Nematoda	—	AF339577	AF339527	—	—	—	Sung et al. 2001
*Ophiocordycepsaustralis*	HUA186147	Hymenoptera	—	KC610784	KC610764	KC610734	KF658678	—	Sanjuan et al. 2015
*Ophiocordycepsaustralis*	HUA186097	Hymenoptera	—	KC610786	KC610765	KC610735	KF658662	—	Sanjuan et al. 2015
*Ophiocordycepscurculionum*	OSC 151910	Coleoptera	—	KJ878918	KJ878885	—	KJ878999	—	Quandt et al. 2014
*Ophiocordycepsirangiensis*	NBRC101400	Hymenoptera	JN943335	JN941714	JN941426	—	JN992449	—	Schoch et al. 2012
*Ophiocordycepskimflemingiae*	SC30	Hymenoptera	—	KX713629	KX713622	KX713699	KX713727	—	Araújo et al. 2018
*Ophiocordycepskonnoana*	EFCC 7315	Coleoptera	—	EF468959	—	EF468753	EF468861	EF468916	Mongkolsamrit et al. 2019
*Ophiocordycepslongissima*	TNS F18448	Hemiptera	—	KJ878925	KJ878892	KJ878971	KJ879005	—	Quandt et al. 2014
*Ophiocordycepsmelolonthae*	OSC.110993	Coleoptera	—	DQ522548	DQ518762	DQ522331	DQ522376	—	Mongkolsamrit et al. 2019
***Ophiocordycepsmonticola***	**BPI 634610**	**Orthoptera**	**OQ709246**	—	—	—	—	—	**This Study**
*Ophiocordycepsnigrella*	EFCC 9247	Coleoptera	JN049853	EF468963	EF468818	EF468758	EF468866	EF468920	Mongkolsamrit et al. 2019
*Ophiocordycepsnutans*	OSC 110994	Hemiptera	—	DQ522549	DQ518763	DQ522333	DQ522378	—	Quandt et al. 2014
*Ophiocordycepspulvinata*	TNS-F 30044	Hymenoptera	—	GU904208	—	GU904209	GU904210	—	Kepler et al. 2011
*Ophiocordycepsravenelii*	OSC 151914	Coleoptera	—	KJ878932	—	KJ878978	KJ879012	KJ878950	Quandt et al. 2014
*Ophiocordycepssinensis*	EFCC 7287	Lepidoptera	JN049854	EF468971	EF468827	EF468767	EF468874	EF468924	Quandt et al. 2014
*Ophiocordycepsstylophora*	OSC_111000	Coleoptera	JN049828	DQ522552	DQ518766	DQ522337	DQ522382	DQ522433	Quandt et al. 2014
*Ophiocordycepsvariabilis*	OSC 111003	Diptera	—	EF468985	EF468839	EF468779	EF468885	EF468933	Mongkolsamrit et al. 2019
*Ophiocordycepsvariabilis*	ARSEF 5365	Diptera	—	DQ522555	DQ518769	DQ522340	DQ522386	DQ522437	Mongkolsamrit et al. 2019
*Paraisariaalba*	HKAS_102484	Orthoptera	MN947219	MN943843	MN943839	MN929085	MN929078	MN929082	Wei et al. 2021
*Paraisariaamazonica*	HUA 186143	Orthoptera	—	KJ917562	KJ917571	KM411989	KP212902	KM411982	Sanjuan et al. 2015
*Paraisariaamazonica*	HUA 186113	Orthoptera	—	KJ917566	KJ917572	—	KP212903	KM411980	Sanjuan et al. 2015
*Paraisariaarcta*	HKAS_102553	Lepidoptera	MN947221	MN943845	MN943841	MN929087	MN929080	—	Wei et al. 2021
*Paraisariaarcta*	HKAS 102552	Lepidoptera	MN947220	MN943844	MN943840	MN929086	MN929079	MN929083	Wei et al. 2021
*Paraisariablattarioides*	HUA186093	Blattodea	—	KJ917559	KJ917570	KM411992	KP212910	—	Sanjuan et al. 2015
*Paraisariablattarioides*	HUA 186108	Blattodea	—	KJ917558	KJ917569	—	KP212912	KM411984	Sanjuan et al. 2015
***Paraisariacascadensis***	**OSC-M-052010**	**Orthoptera**	**OQ709237**	**OQ800918**	**OQ708931**	**OR199814**	**OR199828**	**OR199838**	**This Study**
***Paraisariacascadensis***	**OSC-M-052012**	**Orthoptera**	**OQ709239**	**OQ800920**	**OQ708933**	**OR199816**	**OR199830**	—	**This Study**
***Paraisariacascadensis***	**OSC-M-052017**	**Orthoptera**	**OQ709240**	**OQ800921**	**OQ708934**	**OR199817**	**OR199831**	—	**This Study**
*Paraisariacoenomyia*	NBRC 106964	Diptera	AB968397	AB968385	AB968413	AB968571	—	AB968533	Ban et al. 2015
*Paraisariacoenomyia*	NBRC 108993	Diptera	AB968396	AB968384	AB968412	AB968570	—	AB968532	Ban et al. 2015
*Paraisariagracilioides*	HUA186095	Coleoptera	—	KJ917556	—	KM411994	KP212914	—	Sanjuan et al. 2015
*Paraisariagracilioides*	HUA 186092	Coleoptera	—	KJ917555	KJ130992	—	KP212915	—	Sanjuan et al. 2015
*Paraisariagracilis*	EFCC 3101	Lepidoptera	—	EF468955	EF468810	EF468750	EF468858	EF468913	Sung et al. 2007
*Paraisariagracilis*	EFCC 8572	Lepidoptera	JN049851	EF468956	EF468811	EF468751	EF468859	EF468912	Ban et al. 2015
*Paraisariaheteropoda*	OSC 106404	Hemiptera	—	AY489690	AY489722	AY489617	AY489651	—	Quandt et al. 2014
*Paraisariaheteropoda*	EFCC 10125	Hemiptera	JN049852	EF468957	EF468812	EF468752	EF468860	EF468914	Quandt et al. 2014
*Paraisariaheteropoda*	NBRC 100643	Hemiptera	—	JN941719	JN941422	AB968595	JN992453	AB968556	Ban et al. 2015
*Paraisariaheteropoda*	BCC 18235	Hemiptera	—	JN941720	JN941421	AB968594	JN992454	AB968555	Ban et al. 2015
	(NBRC 100642)								
*Paraisariaheteropoda*	BCC 18246	Hemiptera	AB968411	AB113352	—	MK214083	MK214087	—	Ban et al. 2015
	(NBRC 33060)								
***Paraisariainsignis***	**OSC.164134**	**Coleoptera**	**OQ709231**	**OQ800911**	**OQ708924**	**OR199807**	**OR199822**	—	**This Study**
***Paraisariainsignis***	**OSC.164135**	**Coleoptera**	**OQ709232**	**OQ800912**	**OQ708925**	**OR199808**	**OR199823**	—	**This Study**
***Paraisariainsignis***	**OSC.164137**	**Coleoptera**	**OQ709233**	**OQ800913**	**OQ708926**	**OR199809**	**OR199824**	—	**This Study**
***Paraisariainsignis***	**OSC-M-052004**	**Coleoptera**	**OQ709234**	**OQ800914**	**OQ708927**	**OR199810**	—	—	**This Study**
***Paraisariainsignis***	**OSC-M-052008**	**Coleoptera**	**OQ709236**	**OQ800917**	**OQ708930**	**OR199813**	**OR199827**	—	**This Study**
***Paraisariainsignis***	**OSC-M-052013**	**Coleoptera**	**OQ709244**	**OQ800924**	**OQ708938**	**OR199820**	**OR199834**	—	**This Study**
*Paraisariaorthopterorum*	BBC 88305	Orthoptera	MH754742	—	MK332583	MK214080	MK214084	—	Mongkolsamrit et al. 2019
*Paraisariaorthopterorum*	TBRC 9710	Orthoptera	MH754743	—	MK332582	MK214081	MK214085	—	Mongkolsamrit et al. 2019
*Paraisariaphuwiangensis*	TBRC 9709	Coleoptera	MK192015	—	MK192057	MK214082	MK214086	—	Mongkolsamrit et al. 2019
*Paraisariaphuwiangensis*	BBH 43492	Coleoptera	MH188541	—	MH201169	MH211355	MH211352	—	Mongkolsamrit et al. 2019
***Paraisariapseudoheteropoda***	**OSC-M-052005**	**Hemiptera**	—	**OQ800915**	**OQ708928**	**OR199811**	**OR199825**	**OR199836**	**This Study**
***Paraisariapseudoheteropoda***	**OSC-M-052007**	**Hemiptera**	**OQ709235**	**OQ800916**	**OQ708929**	**OR199812**	**OR199826**	**OR199837**	**This Study**
***Paraisariapseudoheteropoda***	**OSC-M-052022**	**Hemiptera**	**OQ709245**	**OQ800925**	**OQ708939**	**OR199821**	**OR199835**	**OR199841**	**This Study**
***Paraisariapseudoheteropoda***	**OSC-M-052020**	**Hemiptera**	**OQ709243**	**OQ800923**	**OQ708937**	**OR199819**	**OR199833**	—	**This Study**
***Paraisariapseudoheteropoda***	**OSC-M-052009**	**Hemiptera**	**OQ709241**	**OQ800922**	**OQ708935**	**OR199818**	**OR199832**	**OR199840**	**This Study**
*Paraisariarosea*	HKAS_102546	Coleoptera	MN947222	MN943846	MN943842	MN929088	MN929081	MN929084	Wei et al. 2021
***Paraisaria* sp.**	**OSC-M-052011**	**Insecta**	**OQ709238**	**OQ800919**	**OQ708932**	**OR199815**	**OR199829**	**OR199839**	**This Study**
***Paraisaria* sp.**	**OSC-M-052026**	**Insecta**	**OQ709242**	—	**OQ708936**	—	—	—	**This Study**
*Paraisariatettigonia*	GZUH CS14062709	Orthoptera	KT345954	KT345955	—	KT375440	KT375441	—	Wen et al. 2016
*Paraisariayodhathaii*	BBH 43163	Coleoptera	MH188539	—	MK332584	MH211353	MH211349	—	Mongkolsamrit et al. 2019
*Paraisariayodhathaii*	TBRC 8502	Coleoptera	MH188540	—	MH201168	MH211354	MH211350	—	Mongkolsamrit et al. 2019
*Perennicordycepscuboideus*	CEM 1514	Coleoptera	—	KF049609	KF049628	KF049683	—	—	Kepler et al. 2013
*Perennicordycepsprolifica*	TNS-F-18547	Hemiptera	KF049660	KF049613	KF049632	KF049687	KF049649	KF049670	Kepler et al. 2013
*Pleurocordycepsnipponicus*	BCC_2325	Neuroptera	KF049665	KF049622	KF049640	KF049696	KF049655	KF049677	Kepler et al. 2013
*Pleurocordycepssinensis*	ARSEF_1424	Coleoptera	KF049661	KF049615	AY259544	DQ118754	DQ127245	KF049671	Kepler et al. 2013
*Pleurocordycepsyunnanensis*	NBRC 101760	Hemiptera	MN586827	MN586818	MN586836	MN598051	MN598042	MN598060	Wang et al. 2021
*Polycephalomycesformosus*	CGMCC_5.2204	Coleoptera	MN586831	MN586821	MN586839	MN598054	MN598045	MN598061	Wang et al. 2021
*Polycephalomycesformosus*	CGMCC_5.2208	Coleoptera	MN586835	MN586825	MN586843	MN598058	MN598049	MN598065	Wang et al. 2021
*Purpureocilliumatypicola*	CEM 1185	Araneae	—	KJ878907	KJ878872	KJ878955	—	—	Quandt et al. 2014
*Purpureocilliumatypicola*	OSC 151901	Araneae	—	KJ878914	KJ878880	KJ878961	KJ878994	—	Quandt et al. 2014
*Purpureocilliumtakamizusanensis*	NHJ_3497	Hemiptera	—	EU369096	EU369033	EU369014	EU369053	EU369074	Johnson et al. 2009
*Tolypocladiumcapitatum*	OSC 71233	Fungi (Eurotiales)	—	AY489689	AY489721	AY489615	AY489649	DQ522421	Spatafora et al. 2007
*Tolypocladiuminflatum*	OSC 71235	Coleoptera	JN049844	EF469124	EF469077	EF469061	EF469090	EF469108	Kepler et al. 2012
*Tolypocladiumophioglossoides*	OSC 106405	Fungi (Eurotiales)	—	AY489691	AY489723	AY489618	AY489652	DQ522429	Castlebury et al. 2004
*Tolypocladiumnjaponicum*	OSC 110991	Fungi (Eurotiales)	JN049824	DQ522547	DQ518761	DQ522330	DQ522375	DQ522428	Quandt et al. 2014
*Torrubiellomyceszombiae*	NY04434801	Fungi (Hypocreales)	—	ON493543	ON493602	ON513396	ON513398	ON513402	Araújo et al. 2022

### ﻿Data analysis

Sequences derived from the SSU, LSU, TEF, RBP1, RPB2, and ITS were aligned with MUSCLE 5.1 (Edgar 2004). Ambiguous and phylogenetically uninformative regions were manually removed and the trimmed alignments were concatenated for analysis using Geneious Prime® 2023.0.4. A Maximum Likelihood Tree was made using the GTR+I+A algorithm and 1000 bootstrap replicates.

### ﻿Chemical extraction and LCMS analysis

Excisions (0.4–6.7 mg) were made from the endosclerotia of nineteen dried *Paraisaria* collections, individually placed in MeOH (1 ml, HPLC-grade), sonicated for 5 min, and extracted for 1 hr at 35 °C, then 24 h at ambient temperature. The twenty separate extracts were filtered through syringe filters (0.2 µm PTFE) and dried *in vacuo* before dissolution in MeOH (0.1 mg/ml, LC-MS-grade) for analysis by LC-MS, injecting 3 µl on a Phenomenex Kinetex column (2.6 µm C18 100 Å, 50 × 2.1 mm), with H_2_O + 0.1% Formic Acid (A) MeCN + 0.1% Formic Acid (B) as mobile phase solvents at 0.4 ml/min. The LC method was as thus: 0.5 mins at 20% B, a linear gradient from 20–90% B over 14 mins, 4 min at 90% B, a linear gradient from 90–100% B over 0.5 mins, 4.5 mins at 100% B, followed by a linear return to 20% B over 3 mins, and re-equilibration at 20% B for 5 mins, before the next injection. High resolution (Agilent 6545 QToF) mass data were acquired for 26 mins from *m/z* 100–3200, with MS/MS spectra obtained using data-dependent ion selection for up to five precursor ions per duty cycle, excluding precursor ions with *m/z* less than 210, and fragmenting with collision energies of 20, 40, and 60 eV. LCMS data files were converted to mzML format and deposited on the public repository MassIVE (MSV000092591). Extracted ion chromatograms were produced for *m/z* 690–875, corresponding to the mass range for the paraisariamide peptide family (Tehan 2022).

### ﻿Molecular networking

Unprocessed LC-MS files were converted to mzML format and uploaded to the GNPS online molecular networking platform (version 30) (Wang et al. 2016) using the default network settings but with minimum peak intensity set to 3000. The resulting network was downloaded as a graphML file, analyzed, and visualized using (Ctyoscape ver. 3.9.1). The GNPS job is accessible at https://gnps.ucsd.edu/ProteoSAFe/status.jsp?task=6bd4f858a8704e3fa98cb0c66de02248.

### ﻿Principal component analysis

LC-MS data were processed in MZmine v2.53 (Pluskal et al. 2010). Feature detection was performed with noise level set to 1×10^4^. Chromatograms were built using a minimum group size of 5, group intensity threshold set to 1×10^2^, minimum highest intensity set to 2×10^4^, and *m/z* tolerance was set to *m/z* 0.001 or 10 ppm. Chromatogram deconvolution was performed with minimum peak height set to 1×10^4^, peak duration was set to 0.1–10 mins, and the baseline was set to 5×10^2^. Isotope peaks were grouped with mass tolerance *m/z* 0.001 or 15 ppm, RT tolerance was set to 1, with the most intense ion taken as the representative, and max charge was set to 2. Peaks were aligned with mass tolerance *m/z* 0.001 or 12 ppm, RT tolerance set to 0.8 mins, with *m/z* weighted 75% and RT weighted 25%. Feature list rows were filtered for features falling within the range *m/z* 690–875, and RT 5–14 mins, with a minimum of 2 peaks per row, and a minimum of 2 peaks in an isotopic pattern. Gap filling was performed with an intensity tolerance of 10%, mass tolerance *m/z* 0.001 or 15 ppm, and RT tolerance 0.6 mins. The resulting feature list was subjected to Principal Component Analysis (PCA).

## ﻿Results

### ﻿Molecular phylogeny

**Figure 1.**
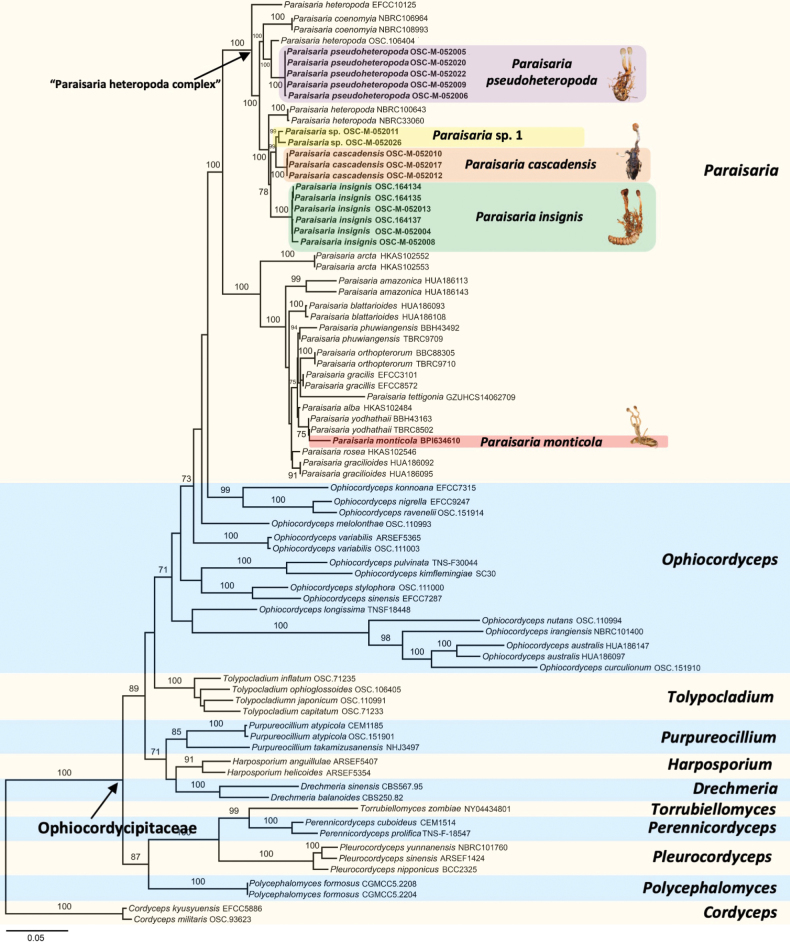
Maximum likelihood tree based on the combined dataset of SSU, LSU, TEF, RPB1, RPB2, and ITS sequences displaying the relationship of *Paraisaria* species within family Ophiocordycipitaceae.

We generated 82 new sequences (16 SSU, 16 LSU, 15 TEF, 14 RPB1, 6 RPB2, and 16 ITS). The combined dataset of 79 taxa afforded a concatenated multi-locus alignment comprising 5,317 bp (1,030 SSU, 955 LSU, 977 TEF, 702 RPB1, 1,037 RPB2, 616 ITS) which was deposited on TreeBASE (accession URL: http://purl.org/phylo/treebase/phylows/study/TB2:S30820). In the resulting phylogenetic tree (Fig. 1), ten genera in the family Ophiocordycipitaceae are represented. *Cordycepskyushuensis* and *C.militaris* (Cordycipitaceae) were designated as outgroup taxa. All genera, with the exception of *Ophiocordyceps*, are supported as monophyletic clades. A clade comprising several species morphologically similar to the well-known cicada pathogen, *P.heteropoda*, referred to here as the “P.heteropoda complex”, is resolved into five well-defined species as well as additional samples revealing cryptic diversity. Two new species within the *P.heteropoda* complex, *Paraisariacascadensis* and *Paraisariapseudoheteropoda*, are supported as monophyletic clades, and are described below. *Ophiocordycepsinsignis* samples produced a monophyletic clade within the *P.heteropoda* complex supporting its combination into *Paraisaria*, and is redescribed based on a fresh collection, which is designated here as an epitype. The type collection of *Ophiocordycepsmonticola* also occurred within the genus *Paraisaria*, grouping closely with *P.yodhathaii* and *P.alba*. It was the only North American *Paraisaria* species analyzed in this study which did not fall within the *P.heteropoda* complex.

### ﻿LC-MS analysis

**Figure 2.**
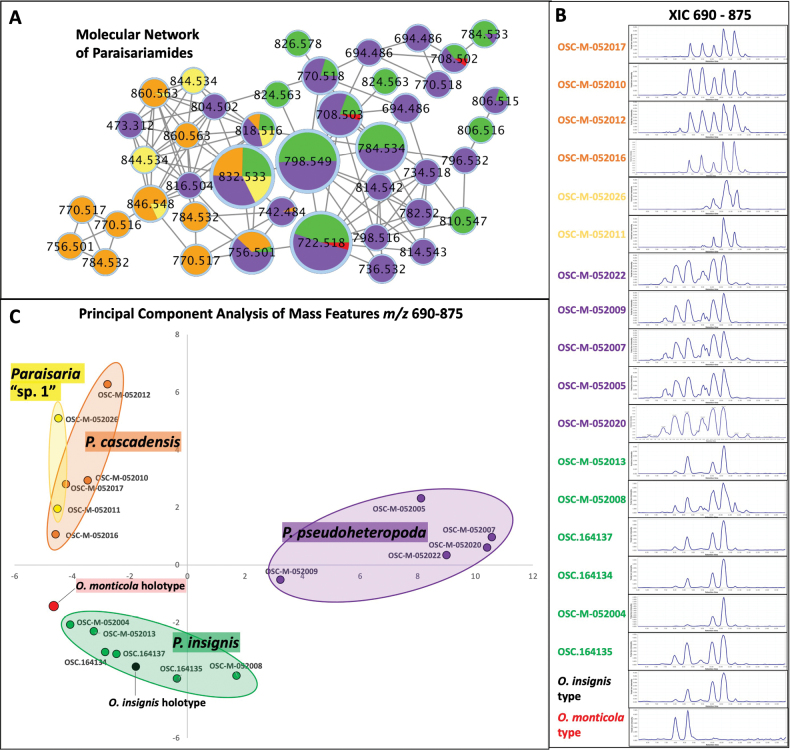
Chemical comparison of paraisariamide content in the endosclerotia of *Paraisaria* species collected in the USA **A** molecular network of the paraisariamide molecular family of cyclic peptides detected in methanol extracts of endosclerotia of *Paraisaria* specimens. Nodes are displayed as pie charts conveying the relative abundance of paraisariamide mass ion features in each *Paraisaria* species (Orange = *P.cascadensis*, Purple = *P.pseudoheteropoda*, Green = *P.insignis*, Yellow = “*Paraisaria* sp. 1”, Red = *P.monticola*) **B** extracted ion chromatograms of *m/z* 690–875 for methanol extracts of endosclerotia of *Paraisaria* specimens **C** principal component analysis of mass features *m/z* 690–875 from methanol extracts of endosclerotia of *Paraisaria* specimens, color-coded by phylogenetic clade.

Molecular Network Analysis of nineteen *Paraisaria* endosclerotium extracts revealed a prominent subnetwork identified as the paraisariamide family of cyclopeptides, with constituent molecular ion masses ([M+H]^+^) ranging from *m/z* 694.49–860.56 (Fig. 2A.). All endosclerotium extract samples were observed to possess a subset of paraisariamide congeners with partial overlap between species. Production of paraisariamide cyclopeptides in host/endosclerotium is thus supported as a conserved chemotype for *Paraisaria*. Paraisariamides can thus potentially be used as a generic diagnostic character. Chromatograms generated from the extracted ion range *m/z* 690–875, corresponding to the mass range for the peptide family of paraisariamides, were unique to and consistent within each species (Fig. 2B). From the processed mass data, a feature list was produced comprising 59 LC-MS ion features (Suppl. material 1). A PCA plot generated from this feature list afforded three major clusters (Fig. 2C). Samples derived from *P.insignis* and *P.pseudoheteropoda* were resolved in distinct clusters. Samples derived from *P.cascadensis* together with samples from its sister clade, “*Paraisaria* sp. 1”, grouped apart from other samples. *Ophiocordycepsmonticola* afforded two prominent ion peaks with quasimolecular ions, *m/z* 708.502 and 722.518 eluting at 8.0 and 8.7 min respectively, and grouped most closely with *P.insignis* in the PCA plot. Qualitatively, the general shape of ion chromatograms was highly conserved within each species and distinct between species. The resolution of species by LC-MS analysis overall accorded very well with the phylogenetic analysis.

### ﻿Taxonomy

#### ﻿ Paraisaria cascadensis

Taxon classificationFungiHypocrealesOphiocordycipitaceae

Tehan, Dooley & Spatafora sp. nov.

57F98506-DE27-542A-9419-F7EE27C73604

 849757

Fig. 3

##### Type material.

***Holotype*.** U.S.A., Washington. Skamania County, Gifford Pinchot National Forest, Mt. St. Helens, at approximately 46.1771, -121,9224. 1,042 m alt., 9 June 2021, on adult *Cyphoderrismonstrosa* buried in the ground, in mixed coniferous forest comprising *Pinuscontorta*, *Pseudotsugamenziesii*, and *Abies* sp., collected by R. Tehan, C. Dooley (RMT-2021-072, OSC-M-052017, ex-holotype living culture: ARSEF 14609.

**Figure 3.**
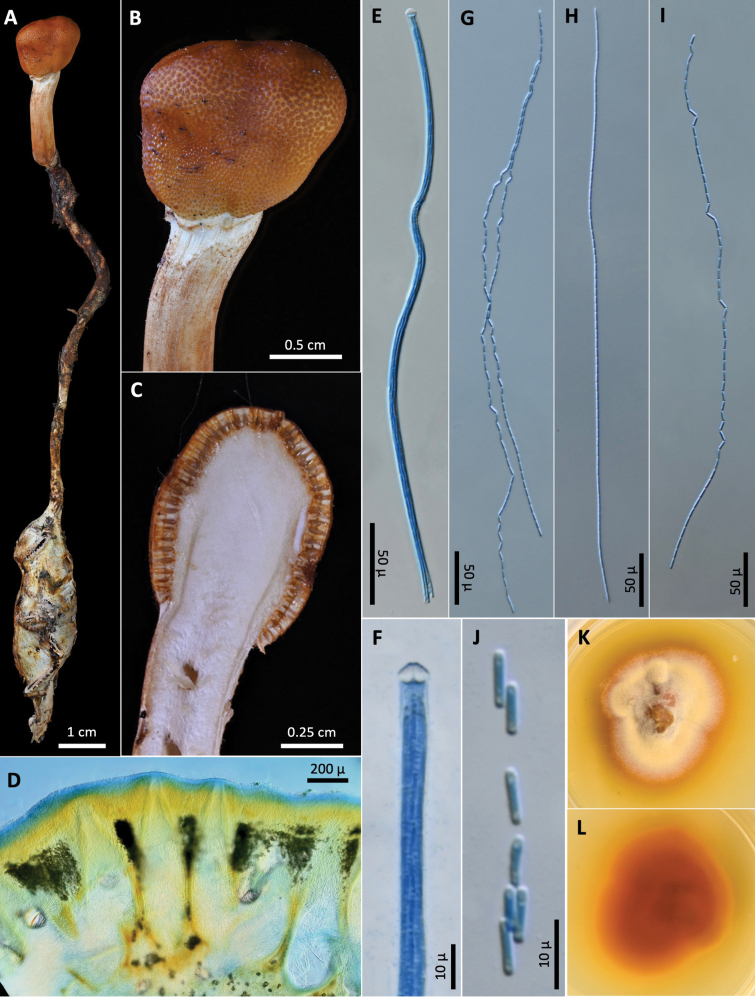
*Paraisariacascadensis***A** OSC-M-052017 **B** fertile head **C** cross section of fertile head showing arrangement of perithecia **D** perithecia **E** ascus **F** Ascus apex **G–I** ascospores **J** part-spores **K, L** colonies on PDA 61 d (**K** obverse, **L** reverse).

##### Etymology.

*cascadensis* occurring in the Cascade Mountain range in the Pacific Northwest, USA.

##### Description.

Stroma capitate, solitary, rhizoids solitary arising from heads of adult *Cyphoderrismonstrosa* buried in soil. Ascogenous portion globose or subglobose, 8–9 × 6–9 mm, chestnut brown. Stipe white to light brown, inside hollow, fibrous, white, 15–17 mm long, 3–4 mm wide, papillate with ostioles of perithecia. Perithecia obclavate, immersed, ordinally arranged, 800–970 × 105–150 µm. Asci hyaline, cylindrical, eight-spored, observed up to 350 µm long × 4.5–7 µm wide, possessing abruptly thickened apex. Ascospores hyaline, filiform, multiseptate, breaking into 64 cylindrical part-spores, (6.3–)7.5–9.5(–10.3) × 1.6–2.2(–2.4) µm.

##### Culture characteristics.

Colonies on PDA 61 days at 20 °C, 28 mm, white to yellow, reverse reddish brown to orange. Mycelium septate, smooth-walled hyaline. No conidial state was observed.

##### Host.

*Cyphoderrismonstrosa* (Prophalangopsidae, Orthoptera).

##### Habitat.

Specimens occur on hypogeous adult hump-winged grigs, *Cyphoderrismonstrosa*, in coniferous forest.

##### Additional materials examined.

U.S.A., Washington: Skamania County, at approximately 46.177, -121.9167, elevation: 974 m, 29 May 2018, on cf. *Cyphoderrismonstrosa* buried in soil, collected by Josh Grefe (OSC-M-052003). U.S.A., Washington: Chelan County, 47.9761, -120.7811, elevation: 865 m, 15 June 2020, on adult *Cyphoderrismonstrosa*, buried in soil, collected by Daniel Winkler, Hans Drabicki (OSC-M-052010). U.S.A., Washington: Skamania County, at approximately 46.1848, -122.1139, elevation: 12332 m, 12 June 2020, on cf. *Cyphoderrismonstrosa*, collected by Ben McCormick (OSC-M-052012). U.S.A., Washington: Skamania County, Gifford Pinchot National Forest, Mt. St. Helens, at approximately 46.1771, -121,9224. 1,042 m alt., 9 June 2021, on adult *Cyphoderrismonstrosa* buried in soil, in mixed coniferous forest comprising *Pinuscontorta*, *Pseudotsugamenziesii*, and *Abies* sp., collected by Richard Tehan, Connor Dooley (RMT-2021-071, OSC-M-052016).

##### Notes.

This species is uncommon and has thus far only been collected in the Cascade Mountains of Washington State in the vicinity of Mount St. Helens at elevations above 850 m. It might be expected to have a broader range on the basis of the range of its host, *Cyphoderrismonstrosa*, which is known to occur in coniferous forest in several Western U.S. states and Canada (The Orthopterists’ Society 2023).

#### ﻿ Paraisaria pseudoheteropoda

Taxon classificationFungiHypocrealesOphiocordycipitaceae

Tehan & Spatafora sp. nov.

750F4D19-8845-5E24-8571-A2C428296D59

 849758

Fig. 4

##### Type material.

***Holotype*.** U.S.A. Arkansas: Searcy County, Grinder's Ferry, 35.985, -92.732, elevation: 252 m, 15 May 2022, on nymphs of cicadidae (Hemiptera) buried in soil, in near *Quercus* sp., *Carya* sp., and *Juniperusvirginiana*, collected by Kerri McCabe (OSC-M-052022, ex-type culture: ARSEF 14616).

**Figure 4.**
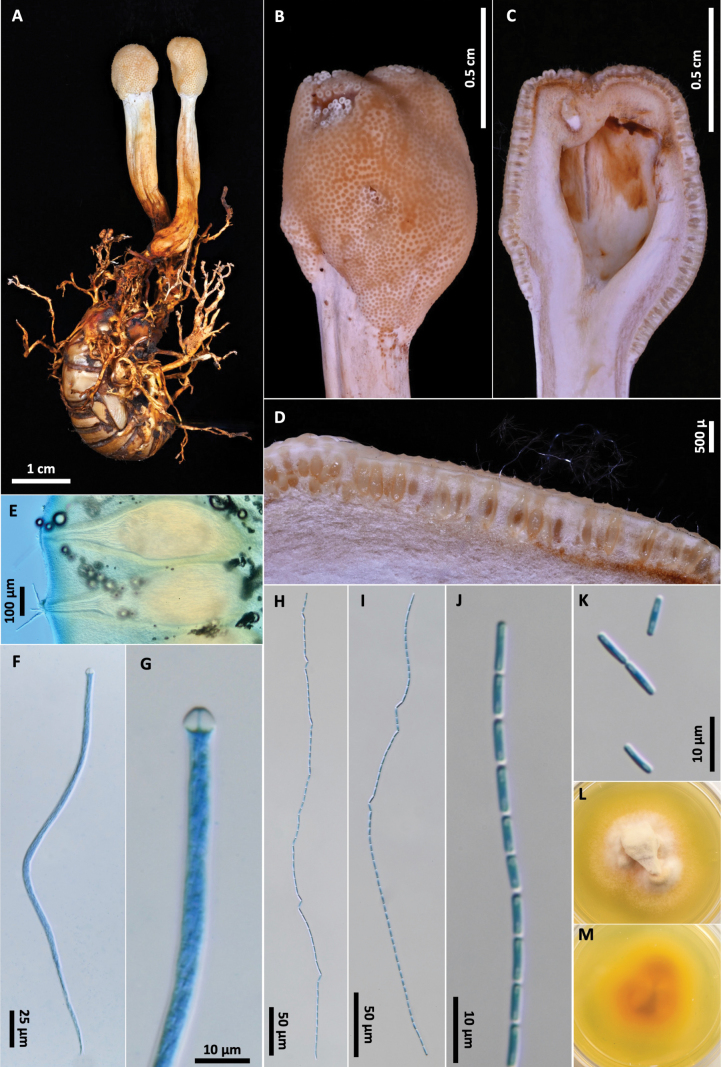
*Paraisariapseudoheteropoda***A** OSC-M-052022 **B** fertile head **C, D** cross section of fertile head showing arrangement of perithecia **E** perithecia **F** ascus **G** ascus apex **H, I** ascospores **J** ascospore tip **K** part-spores **L**, **K** colonies on PDA 61 d (**L** obverse, **M** reverse).

##### Etymology.

*pseudoheteropoda* resembling another cicada-pathogenic species, *Paraisariaheteropoda*.

##### Description.

Stromata capitate or subclavate, unbranched, growing singly or up to two stromata attached by rhizoids to hypogeous nymphs of Cicadidae (Hemiptera). Ascogenous portion globose or subglobose, 9–11 × 7–8 mm, cream to chestnut brown. Stipe white to light brown, inside fibrous, white, 20–53 mm long, 4–5 mm wide, papillate with ostioles of perithecia. Perithecia obclavate, immersed, ordinally arranged 680–745(–760) × (310–)330–420 µm. Asci hyaline, cylindrical, eight-spored, observed up to 420 µm long × 5.5–6.5 µm wide, possessing abruptly thickened apex. Ascospores hyaline, filiform, multiseptate, breaking into 64 cylindrical part-spores, (5.6–)6.2–7.9(–8.7) × 1.6–2.1(–2.4) µm.

##### Culture characteristics.

Colonies on PDA 61 days at 20 °C, 29 mm, white, reverse yellow to orange. Mycelium septate, smooth-walled hyaline. No conidial state was observed.

##### Host.

Nymphs of Cicadidae (Hemiptera).

##### Habitat.

Specimens occur on hypogeous nymphs of cicadae at the base of coniferous and deciduous trees, especially oaks.

##### Additional materials examined.

U.S.A. Missouri: Barry County, Cassville, at approximately 36.5586, -93.6833, elevation: 301 m, 26 May 2019, on nymph of cicada buried in soil, collected by Aaron Peters, (OSC-M-052005) U.S.A. Missouri: Barry County, Cassville, at approximately 36.6501, -93.7031, elevation: 382 m, 16 May 2019, on nymph of cicada buried in soil, collected by Aaron Peters (OSC-M-052007) U.S.A. Missouri: Barry County, Cassville, at approximately 36.5586, -93.6833, elevation: 301 m, 4 April 2020, on nymph of cicada buried in soil, collected by Aaron Peters (OSC-M-052009, living culture: ARSEF 14610). U.S.A. Kentucky: Lincoln County, Crab Orchard, at approximately 36.464, -84.51, elevation: 290 m, 19 April 2021, on nymph of cicada buried in soil, collected by Michael Roberts (OSC-M-052015). U.S.A. Tennessee: Putnam County, Cookerville, at approximately 36.163, -85.501, elevation: 337 m, 17 April 2022, on nymph of cicada buried in soil in mixed hardwood forest comprising *Quercus* sp., *Fagus* sp., *Populus* sp. and *Arundinariagigantea*, collected by Jamie Newman (OSC-M-052019). U.S.A. Tennessee: Putnam County, Silver Point, at approximately 36.1409, -85.7374, elevation: 180 m, 17 April 2022, on nymph of cicada buried in soil among *Acernegundo*, *Carpinuscaroliniana*, *Carya* sp., *Quercusrubra*, *Lindera* sp., *Amphicarpaeabracteata*, *Phloxdivaricata*, *Salvialyrata*. collected by Holly Taylor (OSC-M-052020). U.S.A. Arkansas: Searcy County, Grinder's Ferry, at approximately 35.983, -92.719, elevation: 222 m, 14 May 2022, on nymphs of cicadae buried in soil, in near *Quercus* sp., Carya sp., and *Juniperusvirginiana*, collected by Kerri McCabe (OSC-M-052021). U.S.A. Missouri: Barry County, Roaring River, at approximately 36.5593, -93.683, elevation: 296 m, 24 May 2022, on nymphs of cicadae buried in soil, collected by Aaron Peters, (OSC-M-052023). U.S.A. Virginia: Albemarle County, Charlottesville, at approximately 38.0812, -78.4657, elevation: 133 m, 31 May 2022, on nymph of cf. *Neotibicen* sp. (Cicadidae, Hemiptera) buried in soil near *Acerrubrum*, collected by Amelio Little (OSC-M-052024). U.S.A. Missouri: Barry County, Roaring River, at approximately 36.5583, -93.6836, elevation: 305 m, 25 May 2022, on nymphs of cicadae buried in soil, collected by Aaron Peters, (OSC-M-052025). U.S.A. Alabama: St. Clair County, Leeds, at approximately 33.5540, -86.5382, elevation: 198 m, 12 March 2023, on nymphs of cicadae buried in soil, collected by Courtney Mynick, (OSC-M-053266). U.S.A. Alabama: Jefferson County, Birmingham, at approximately 33.4402, -86.8894, elevation: 195 m, 16 March 2023, on nymphs of cicadae buried in soil, collected by Bucky Raeder, (OSC-M-053267).

##### Notes.

This species is the only *Paraisaria* species known to occur on cicadas in North America. In morphology and geographic distribution, it overlaps with *P.insignis* but that species is distinguished by its strict occurrence on Coleoptera. *P.pseudoheteropoda* sometimes has a pallid stroma which is not observed in *P.insignis*.

#### ﻿ Paraisaria insignis

Taxon classificationFungiHypocrealesOphiocordycipitaceae

(Cooke & Ravenel) Tehan & Spatafora comb. nov.

CC3AFBAB-C7B9-5F63-961D-36D3CF2B5656

 849763

Fig. 5

Cordyceps insignis Cooke & Ravenel, Grevillea 12(no. 61): 38 (1883). Basionym.
Ophiocordyceps insignis (Cooke & Ravenel) G.H. Sung, J.M. Sung, Hywel-Jones & Spatafora, *Stud. Mycol*. 57: 43 (2007). Synonym.

##### Type.

U.S.A. South Carolina, “seaboard”, 4 January 1881, on larva coleoptera, collected by H. W. Ravenel. (Holotype: Ravenel 3251, K-M 1434269).

**Figure 5.**
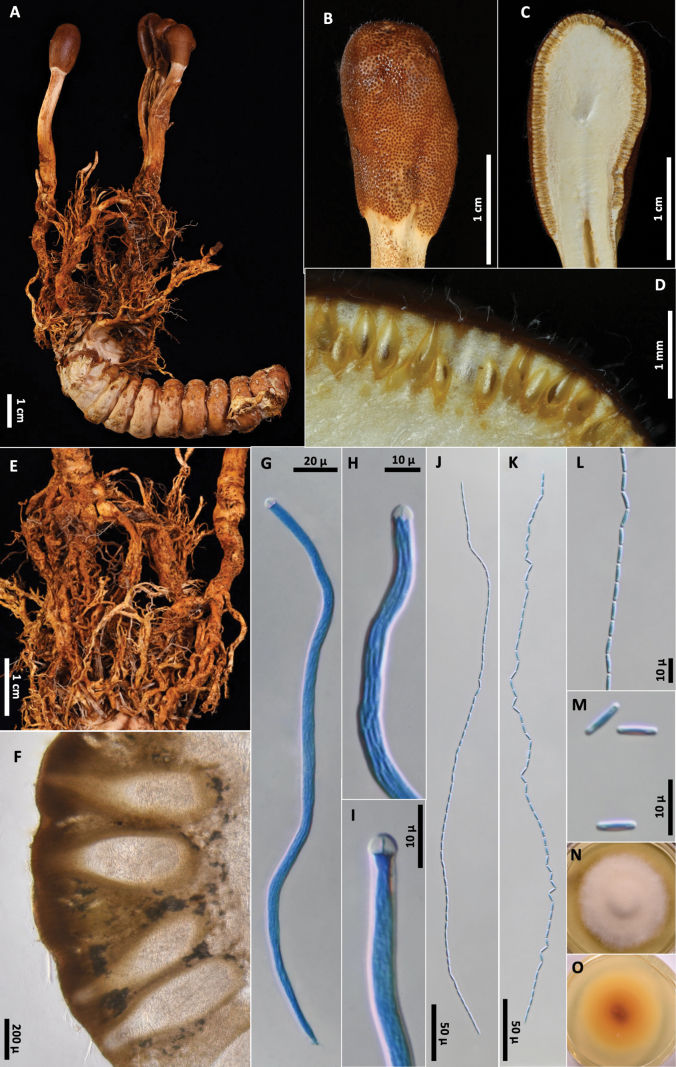
*Paraisariainsignis***A** OSC-M-052013 Epitype**B** fertile head **C, D** cross section of fertile head showing arrangement of perithecia **E** rhizomorphs **F** perithecia **G** ascus **H, I** asci apices **J–L** ascospores **M** part-spores **N, O** colony on PDA 70 d (**N** obverse, **O** reverse).

***Epitype*** designated here: U.S.A. Arkansas: Saline County, Avilla, at approximately 34.713, -92.587, elevation: 169 m, 2 April 2021, on larva of *Prionusimbricornis* (Cerambycidae, Coleoptera) buried in soil near *Quercus* sp., collected by Jay Justice (OSC-M-052013, ex-type living culture ARSEF 14611).

##### Description.

Stromata capitate, unbranched, growing singly to gregarious, in groups of up to four stromata on a single host. Stromata 20–52.5 mm long. Ascogenous portion brown, globose to oblong, 8–22 mm long × 7–16 mm wide, papillate with ostioles of perithecia. Stipe golden yellow to reddish orange, sometimes furfuraceous toward upper half, 14–25 × 4–9 mm long, attached to hypogeous host by thick mats of fibrous, tangled, yellow to reddish orange rhizomorphs, extending 25–45 mm. Mycelial growth occurring between, and sometimes over, larval segments, forming a thin membrane. Perithecia embedded, obclavate, brown, (520–)640–800(840) × (160–)185–250(–270) µm. Asci hyaline, cylindrical, up to 380 µ long × (3.8–)4.0–5.9(–7.5) µm, possessing abruptly thickened apex. Ascospores hyaline, filiform, smooth, disarticulating into 64 part-spores. Part-spores, cylindrical, 6.3–9.0(–10.5) × 2.5–3.5 µm. Growing on larvae of Prionuscf.imbricornis. (Cerambycidae, Coleoptera).

##### Culture characteristics.

Colonies on PDA 70 days at 20 °C, 37.5 mm, white, reverse reddish brown to yellow. Mycelium septate, smooth-walled hyaline. No conidial state was observed.

##### Host.

larvae of Prionuscf.imbricornis. (Cerambycidae, Coleoptera)

##### Habitat.

Specimens occur on hypogeous larvae of coleoptera typically at the base of oak trees.

##### Additional materials examined.

U.S.A. Arkansas: Saline County, Avilla, at approximately 34.713, -92.587, elevation: 169 m, 18 March 2018, on larva of *Prionusimbricornis* (Cerambycidae, Coleoptera) buried in soil near *Quercus* sp., collected by Jay Justice (OSC.164134). U.S.A. Arkansas: Saline County, Avilla, at approximately 34.713, -92.587, elevation: 169 m, 2 April 2018, on larva of *Prionusimbricornis* (Cerambycidae, Coleoptera) buried in soil near *Quercus* sp., collected by Jay Justice (OSC.164135, living culture: ARSEF 14615). U.S.A. Arkansas: Saline County, Avilla, at approximately 34.713, -92.587, elevation: 169 m, 21 April 2018, on larva of *Prionusimbricornis* (Cerambycidae, Coleoptera) buried in soil near *Quercus* sp., collected by Jay Justice (OSC.164136). U.S.A. Arkansas: Pulaski County, North Little Rock, at approximately 34.7989, -92.312, elevation: 99 m, 17 April 2018, on larva of *Prionusimbricornis* (Cerambycidae, Coleoptera) buried in soil near *Quercus* sp., and *Ulmus* sp., collected by Sheila Griffin (OSC.164137). U.S.A. Missouri: Barry County, Cassville, at approximately 36.6116, -93.6938, elevation: 381 m, 16 April 2019, on larva of *Prionusimbricornis* (Cerambycidae, Coleoptera) buried in soil, collected by Aaron Peters (OSC-M-052004). U.S.A. TEXAS: Harris County, Friendswood, at approximately 29.5501, -95.1972, 19 m, 15 February 2020, on larva of Coleoptera, cf. *Prionusimbricornis* buried in soil, collected by Brett Jackson (OSC-M-052008). U.S.A. Mississippi: Otibbeha County, at approximately 33.4576, -88.7859, elevation: 109 m, 29 March 2021, on larva of Coleoptera buried in soil near *Quercus* sp., collected by Carol Siniscalchi (OSC-M-052014) U.S.A. Arkansas: Saline County, Avilla, at approximately 34.713, -92.587, elevation: 169 m, 21 April 2018, on larva of *Prionusimbricornis* (Cerambycidae, Coleoptera) buried in soil near *Quercus* sp., collected by Jay Justice (OSC-M-052018, living culture: ARSEF 14617). U.S.A. Georgia: Greene County, Greensboro, at approximately 33.556, -83.262, elevation 152 m, 25 March 2023, on larva of coleoptera, buried in soil, collected by Patti Chaco (OSC-M-053264). U.S.A. Georgia: Bibb County, Musella, at approximately 32.8491, -83.8886, elevation 145 m, 2 April 2023, on larva of coleoptera, buried in soil near *Quercusphellos*, collected by Rose Payne (OSC-M-053265).

##### Notes.

Recent collections of this species were initially determined to not match any described species and were given the provisional name *Paraisariatortuosa*, which was used in a doctoral dissertation (Tehan 2022), and in conference presentations. The conspecificity with *Ophiocordycepsinsignis* (=*Cordycepsinsignis*) was considered but it was difficult to reconcile Cooke’s description of the stroma as “livid purple”. However, that species was described from a dried specimen and the true colors of the fresh specimen were evidently not observed by the authority. Petch (1935) cast doubt on the accurate description of the color of *C.insignis* and though the original host is not able to be precisely identified, Petch’s analysis here is helpful, suggesting based on morphology that the host is one that pupates in wood, which accords with the host of recent collections identified as *Prionusimbricornis*. Ultimately, chemical comparison of fresh collections to the holotype was definitive in the identification of the fresh collections, and strongly supports the combination into *Paraisaria*.

#### ﻿ Paraisaria monticola

Taxon classificationFungiHypocrealesOphiocordycipitaceae

(Mains) Tehan & Spatafora comb. nov.

42988EA0-BDCE-57A4-95D7-78CC5F23A900

 849764

Fig. 6

Cordyceps monticola Mains, *Mycologia* 32(3): 310 (1940). Basionym.
Ophiocordyceps monticola (Mains) G.H. Sung, J.M. Sung, Hywel-Jones & Spatafora, *Stud. Mycol*. 57: 45 (2007). Synonym.

##### Materials examined.

**Type**: U.S.A. Tennessee, Monroe County, Vonore, June 1936, on adult *Neocurtillahexadactyla*. collected by G. L. Williams. (BPI 634610).

**Figure 6.**
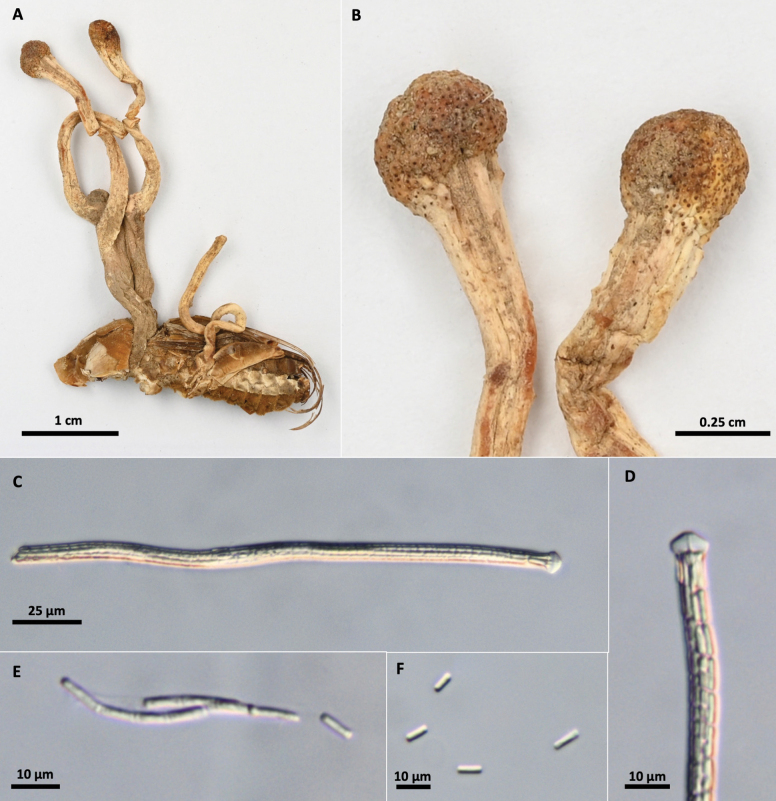
*Paraisariamonticola***A** holotype BPI 634610 **B** fertile head **C** ascus **D** ascus apex **E** portion of ascospore **F** part spores.

##### Notes.

*P.monticola* is known to occur on adult Northern mole cricket, *Neocurtillahexadactyla* (= *Gryllotalpahexadactyla*, Orthoprtera, Gryllotalpidae). Other pathogens of mole crickets, Gryllotalpidae include *Beauveriagryllotalpidicola*, *Beauveriasinensis*, *Cordycepsneogryllotalpae*, *Ophiocordycepsgryllotalpae*, *Ophiocordycepskrachonicola*, and *Polycephalomycsalbiramus*, all of which are only known from east Asia. Lloyd (1920) reported *C.gryllotalpae* from a mole cricket collected in Louisiana, USA, but that specimen was immature, and bore only cylindrical immature stromata with no ascogenous tissue. Owing to the absence of microanatomical character data available for *C.gryllotalpae*, and the lack of genetic data available for either species, future studies could compare *P.monticola* to *C.gryllotalpae* by chemical means, focusing on paraisariamide content of the fungal endosclerotium. *P.monticola* is only known from the type collection.

### ﻿Additional *Paraisaria* specimens examined

Two additional collections were examined which were phylogenetically closest to *P.cascadensis* but occurring on undetermined insect hosts, outside of the known geographic distribution of *Cyphoderrismonstrosa*, the host of *P.cascadensis*. Together they form a clade which is sister to *P.cascadensis*. We do not consider these collections to be conspecific to *P.cascadensis*, but their formal description was not within the scope of the present study owing to lack of adequate sampling and host data. We anticipate that they represent two distinct new species, the description of which requires further sampling. U.S.A., California: Mendocino County, Ukiah, at approximately 39.1568, -123.2328, elevation: 352 m, 5 April 2019, on undetermined insect host buried in soil, collected by Warren Cardimona (OSC-M-052011) U.S.A., Iowa: Johnson County, Solon, at approximately 41.7572, -91.5457, elevation: 238 m, 30 June 2022, on undetermined insect host buried in soil, collected by Ross Salinas (OSC-M-052026).

## ﻿Discussion

In this study, two new *Paraisaria* species are described and two known species are combined into *Paraisaria*. The entomopathogenic fungal genus *Paraisaria* thus currently comprises 18 formally described species which occur on six continents, as deduced from a combination of herbarium records (MycoPortal 2023) and citizen science observations (iNaturalist 2023). The extent of *Paraisaria* diversity both in North America and worldwide is not comprehensively reflected in this study, which warrants future studies of this group. The results of our phylogenetic and chemical analyses support the presence of additional cryptic diversity yet to be elucidated. For such a geographically widespread genus, there has been a relative paucity of sampling and analyses of *Paraisaria* specimens globally. Continued study of this group promises to reveal additional new *Paraisaria* species, each with the potential for new specialized metabolite discovery. In this study, *Paraisaria* populations in North America prove to be enriched in species falling within the *Paraisariaheteropoda* complex. Species in this clade are characterized by fruiting bodies with yellow, brown, and reddish hues and prodigious orange to brown rhizomorphs attaching to hypogeous insect hosts. Aboveground portions of the fruiting bodies in some respects resemble the truffle parasite, *Tolypocladiumcapitatum*, with which they have been compared (Cooke 1883), and with which they are frequently confused. Numerous host shifts have accompanied speciation in the *P.heteropoda* complex with species occurring on insect hosts in orders Hemiptera, Diptera, Coleoptera, and Orthoptera. Host identification is critical for field identification of North American *Paraisaria* species. *P.insignis* and *P.pseudoheteropoda* overlap extensively in fruiting body morphology and geographic distribution but are easily distinguished by their respective distinct hosts. *P.insignis* occurs strictly on coleopteran hosts and *P.pseudoheteropoda* is the only known *Paraisaria* species to occur on cicadas in North America. *P.cascadensis* and *P.monticola* both occur on orthopteran hosts, but the geographic distribution of *P.cascadensis* appears to be restricted to montane regions of the Pacific Northwest, which accords with the distribution of its host, *Cyphoderrismonstrosa*. *P.monticola* is only known from the type specimen collected in Vonore, TN. Re-collection efforts for this species would be valuable and could focus on records of its host *Neocurtillahexadactyla*, in the vicinity of the type locality. Notably, *N.hexadactyla* is widely distributed, and may support a wide distribution of *P.monticola*.

The life cycles of *Paraisaria* species, including mode of infection of their insect hosts, their possible occurrence in soil, as endophytes, saprophytic, and nematophagous nutritional modes, are not well characterized. Owing to the observation that *Paraisaria* species produce fruiting bodies in spring months in North America, we hypothesize that they colonize their insect hosts in the prior season and overwinter as endosclerotia which are observed to possess high concentrations of cyclopeptide specialized metabolites. The molecular structures, biological activities, and chemical ecology of *Paraisaria* specialized metabolites are the focus of ongoing studies (Tehan 2022).

The targeted LC-MS analysis of specialized metabolites from fungi that are only partially represented in phylogenetic analyses represents a robust application of chemotaxonomy to resolve species. Fungi that produce cyclopeptides may be especially good candidates for chemotaxonomic profiling as many cyclopeptides are particularly resistant to degradation by oxidation, heating, or proteolytic cleavage (Haque and Grayson 2020). Chemotaxonomic profiling of stable metabolites also provides a framework for the analysis of fungal groups lacking genetic data for type specimens, whereby type specimens that afford only chemical data can be linked to samples for which both chemical and genetic data are available, if both types of data resolve species groups. The lack of genetic data for type material is especially challenging when type specimens are very old and possess degraded, highly-fragmented DNA, and for which no suitable neotype has been designated. Micromorphological characters lack robustly distinct differences between *Paraisaria* species for use in reliable species diagnoses. It was thus critical to compare chemical profiles of recent collections of *P.insignis* to the holotype to rigorously establish their conspecificity. Conservation of the general paraisariamide chemotype also supports paraisariamides as chemotaxonomic markers for genus *Paraisaria*, as these compounds were detected in the endosclerotia of all *Paraisaria* specimens analyzed. These markers are substantially more durable than DNA over long periods of time as is evident from the definitive detection of these compounds in the 142-year-old holotype of *P.insignis*. Notably, the shape of chromatograms was visually identical between old and new specimens, indicating that even the relative abundance of paraisariamide congeners within a sample is preserved. LC-MS/MS profiling surveys should be conducted across *Paraisaria* species and related groups of fungi to assess the extent of the paraisariamide molecular family and confirm the utility of these metabolites as chemotaxonomic markers.

Other specialized metabolite families may offer promise as critical chemotaxonomic markers, depending on the relative stability of their biosynthetic genes over time, and whether or not they are reliably expressed. For example, genomic analyses show that the cyclosporin genotype is highly conserved within the insect pathogen, *Tolypocladiuminflatum* (Ophiocordycipitaceae), whereas peptaibiotics have evolved rapidly (Olarte et al. 2019) though neither cyclosporins nor peptaibiotics are detected by LCMS in every *Tolypocladium* strain exhibiting those genotypes (Blount 2018; Tehan et al. 2022).

*Ophiocordycepsblattae*, the type species of the large genus *Ophiocordyceps*, presents another system for potential chemotyping to compare with the various paraphyletic clades of *Ophiocordyceps*. Grounding of genus *Ophiocordyceps* in a type species to strictly define a core *Ophiocordyceps* clade and circumscribe other clades, has remained a longstanding problem owing to the rarity of the type species, and age of its holotype specimen. Increasingly routine chemical profiling by high resolution LC-MS and metabolomics analysis applied to the characterization of fungi in taxonomic studies adds an additional layer of phenotypic assessment that could be indispensable for taxon circumscriptions. Increasing efforts to profile and characterize specialized metabolites in fungi will not only provide useful data for taxonomists but is critical for understanding fungal ecology and may also guide pharmaceutical drug discovery efforts. These pursuits are highly complementary, as demonstrated here and in ongoing research. The isolation, structure elucidation, organic synthesis, biosynthesis, biological characterization, and chemical ecology of the paraisariamides are the focus of ongoing research.

## Supplementary Material

XML Treatment for Paraisaria cascadensis

XML Treatment for Paraisaria pseudoheteropoda

XML Treatment for Paraisaria insignis

XML Treatment for Paraisaria monticola

## ﻿Additional information

### Author ORCIDs

Richard M. Tehan https://orcid.org/0000-0001-7039-3610

Connor B. Dooley https://orcid.org/0009-0007-5692-1182

Edward G. Barge https://orcid.org/0000-0001-8473-7867

Kerry L. McPhail https://orcid.org/0000-0003-2076-1002

Joseph W. Spatafora https://orcid.org/0000-0002-7183-1384

### Data availability

All of the data that support the findings of this study are available in the main text or Supplementary Information.

## Supplementary materials

Supplementary material 1 Endosclerotia LCMS feature list Data type csv Explanation note This table comprises processed LCMS data for methanol extracts of the endosclerotia of 19 vouchered specimens. https://binary.pensoft.net/file/935315This dataset is made available under the Open Database License (http://opendatacommons.org/licenses/odbl/1.0/). The Open Database License (ODbL) is a license agreement intended to allow users to freely share, modify, and use this Dataset while maintaining this same freedom for others, provided that the original source and author(s) are credited.Author: Richard M. Tehan
